# Removing records on animals and nonhumans in Ovid Embase: a comparison of 11 filters

**DOI:** 10.5195/jmla.2026.2437

**Published:** 2026-10-01

**Authors:** Helen Fulbright, Connor Evans

**Affiliations:** 1 Primary Contact, helen.fulbright@york.ac.uk, Research Fellow in Information Science / Information Specialist, Centre for Reviews and Dissemination, University of York, York, England, United Kingdom; 2 connor.evans@york.ac.uk, Research Fellow in Evidence Synthesis, Centre for Reviews and Dissemination, University of York, York, England, United Kingdom

**Keywords:** Bibliographic database, Embase, MEDLINE, indexing, information retrieval, search strategies

## Abstract

**Objective:**

To understand and compare the impact of 11 filters to remove records on animals or nonhumans in Ovid Embase, which are routinely used despite there being no performance data for them. To understand the indexing of relevant subject headings in Embase.

**Methods:**

To assess filter performance, we screened and categorized 3,000 records as ‘should be removed' or ‘should be retained' and calculated the sensitivity, specificity, and overall accuracy for each filter. We also reported on the number of records with human participants or data that were incorrectly removed by each filter.

**Results:**

Method 11 was the most sensitive, correctly retaining 90.6% records, whereas method 3 had the highest specificity, correctly removing 71.5% records. Of the records incorrectly removed by each filter, method 11 removed the fewest records with human participants or data at 10.9%, whereas method 3 removed the most at 14.3%. Filters that did not remove records indexed with ‘nonhuman/' had higher sensitivity. Filter performance could vary by subject, publication type and language due to differences in indexing.

**Conclusion:**

In choosing a search filter, information specialists and review teams should discuss whether animals or nonhumans could feature in relevant studies.

## INTRODUCTION

Evidence syntheses interested in humans as a subject or population often remove records on animals and nonhumans where certain studies (e.g., pre-clinical studies using animals) are ineligible for inclusion. These records are removed using search filters – pre-tested search terms designed to filter results to specific criteria such as the population or study type [1]. Filters described as ‘validated’ have been tested against a ‘gold standard’ or set of known relevant and irrelevant records, whereas ‘non-validated’ filters may have undergone limited testing [2]. In Ovid Embase, several non-validated search filters are routinely used to remove records on animals or nonhumans. Although the database contains an inbuilt limit to filter to results indexed with the Emtree subject heading ‘human/’, this risks exclusion of relevant records where this indexing term has not been applied. The same issue on Ovid MEDLINE can be mitigated with a widely used method, applied in the Cochrane highly-sensitive search strategies for randomized controlled trials (RCTs) [3]. This method removes records indexed with subject headings for animals that are not also indexed with humans. It is therefore designed to limit records to those likely to be about humans by excluding records that are *only* about animals [4]. Many routinely used filters designed for this purpose in Ovid Embase also follow this structure.

As filters designed to remove records on animals or nonhumans are rarely credited in published evidence syntheses (including the widely used MEDLINE filter), many of those designed for Ovid Embase are of unknown authorship. Several such filters, which have been routinely used in Ovid Embase, can be found in a diverse range of sources. Some can be found in the appendices of published literature reviews, such as one used by the Cochrane Common Mental Disorders Group (CCMD) [5] and another used by York Health Economics Consortium (YHEC) [6]; some are found in the strategies used to populate the Database of Abstracts of Reviews of Effects (DARE) and the NHS Economic Evaluations Database (NHS EED) [7]; others appear in filter resources by the InterTASC Information Specialists’ Sub-Group [8] and Canada’s Drug Agency [9]; Ovid Embase has its own filter to remove animal or nonhuman records [10]; and another is part of a validated filter to find RCTs in Ovid Embase [11], though we do not have separate data on its performance. The unknown authorship of many of these filters may reflect that these are custom-made, unpublished or non-validated. It also means we do not know the context in which they were developed and their exact intended purpose. There are numerous differences in the design of these filters, with many exclusively using Emtree terms but some also employing the title or abstract fields. There is also variation in the Emtree headings applied as well as other differences in approach.

The challenges of defining and removing records on animals have been highlighted by Finnegan et al. [12]. Issues such as how we define these records mean that understanding the efficacy of filters designed to remove them yields numerous complexities. Firstly, just as we cannot rely on all records that are relevant to humans to be indexed with ‘human/’, we also cannot rely on records relevant to both animals/nonhumans *and* humans to use this indexing term. Therefore, these records could be excluded from search results if desired Emtree terms such as ‘human/’ or ‘human experiment/’ are missing on records with humans as a subject or population. This is important to consider, as animals are involved in human health in animal-assisted therapies; in detecting conditions such as cancer or low blood sugar; as sources of disease transmission or injury; and in various other respects. Similarly, as the Emtree term ‘nonhuman/’ can encompass animals, bacteria, viruses, and plants, we may not consider the full breadth of this term during filter selection, despite the relevance of nonhumans to human health. This prompts us to consider several questions: how are indexers assigning Emtree headings to records; what are information specialists using the filters to remove; what types of animal or nonhuman records do research teams want removed (and how might this vary on a review-by-review basis); and do information specialists and review teams discuss if the use of a filter is appropriate?

The use of search filters to remove records on animals/nonhumans is a subject which has received little attention to date. In 2025, a study by Askin et al on the widely used MEDLINE filter applied in the Cochrane highly-sensitive search strategies, found that records machine-indexed with the subject heading ‘humans/’ were almost twice as likely to be incorrect compared with manual indexing [13]. Prior to this, a poster by Soudant et al. in 2021 questioned the reliability of three non-validated filters to remove animal records in both MEDLINE and Embase, and reported on records with human participants that had been incorrectly removed [14].

As there is no direct performance data on any of the routinely used filters designed for Embase, filter performance and reliability are important considerations. It would be valuable to understand how these perform and compare to each other to inform discussion and decision-making about filter use.

## OBJECTIVES

This is an exploratory study which aims to investigate the impact of 11 filters to remove records on animals or nonhumans in Ovid Embase. The study explores how the filters work and compare to each other to facilitate informed decision making about filter use. The main objectives are to:

- understand how records are indexed with Emtree subject headings and how this could affect filter performance;
- explore how the 11 filters compare in terms of the proportion of records correctly removed (specificity) or retained (sensitivity); and
- explore how many records with human participants or data were incorrectly excluded by each filter.

## METHODS

A summary of the methodology is as follows, we:

- communicated with Elsevier to ask questions on how records are indexed with Emtree subject headings;
- double screened a sample of 3,000 records to judge whether records should be removed or retained, using three separate datasets; and
- determined how the filters compare in their recall of records in the ‘should be removed’ and ‘should be retained’ categories; and in the number of incorrectly removed records with human participants or data.

### 1. Understanding How Records Are Indexed with Emtree Terms

Elsevier’s indexing guide for Embase was used to understand how indexing might affect filter performance. We also e-mailed Elsevier to enquire: if indexers at Elsevier use the scope notes in determining whether to use Emtree subject headings relating to humans, animals, and nonhumans, and the meaning and interpretation of the scope notes for the Emtree term ‘human/’ [15].

Embase records are automatically indexed with Emtree terms based on their titles, abstracts and author keywords [15]. However, records which meet certain criteria (according to an automated process), are then manually indexed using the full text by indexers at Elsevier with a background in biomedicine [15]. This occurs for records with one or more Emtree terms for diseases, drugs, or devices, and records identified as: reviews, short surveys (for case reports only), meta-analyses, and systematic reviews [15]. During manual indexing, indexers at Elsevier use Emtree’s taxonomy as well as some additional rules and internal notes to ensure consistency [16].

Automatic indexing is retained for pre-prints and conference abstracts, and records with the status ‘in press’ or ‘in process’ unless listed as ‘published’ or ‘processed’ respectively [15]. This means there are differences in the indexing of conference abstracts and pre-prints in comparison to other publication types commonly sought in evidence syntheses. Records published in languages other than English are indexed based on the English title and abstract only [15] and do not meet the criteria for manual indexing, except for those in the Embase French Local Literature Module [17].

The scope notes for the Emtree heading ‘human/’, includes wording to describe its application to records ‘where humans are a feature’. Elsevier explained that this could be ‘anything from a human patient in a case report to a rural area, from a major clinical study (human subjects) to an environmental study in an urban area’ [18]. They noted that this wording was chosen ‘to include situations where there is a human presence, without the study necessarily including human subjects’ [18].

### 2. Screening Records on: Animals/Nonhumans; Humans; and Animals/Nonhumans and Humans

To create our datasets, we developed three search strategies for Ovid Embase designed to find records likely to belong to one of the following categories:

1. animals/nonhumans;
2. humans; and
3. animals/nonhumans and humans.

These categories were chosen to provide a more comprehensive set of records than might be retained or removed by the filters compared with searching for topics only relevant to humans. Results for datasets 1 and 3 are not individually reported in this paper but are available in Appendix B. This study focuses on data from the humans dataset (as this is the most representative of topics that would use a filter) as well as all three datasets combined.

Each of the three search strategies included 16 lines of search terms and applied the ‘all fields’ field code. We chose search terms to find records likely to belong to each category only (e.g., veterinary parasitology for the animals/nonhumans category; personality disorder for the humans category; and human-animal bond for the animals/nonhumans and humans category).

The search strategies employed a filter for systematic reviews [7] as well as a filter for RCTs [11], as these study types are commonly sought in evidence syntheses. The study filters were adapted to exclude any lines that removed records on animals or nonhumans. The 11 filters (see Table 1) were then employed consecutively in each of the three search strategies. These were applied with the NOT operator and the AND operator, so we could export records that would be removed or retained by the 11 methods. All lines removing or retaining records were pooled together using the OR operator. For each of the three searches, we used a sample of 500 records removed by the filters and 500 retained by the filters, totalling 1,000 for each category and 3,000 overall. To obtain each of our samples of 500, we used the ‘range’ box on Embase to export a selection of record numbers and ranges, totalling 500. Many of the 11 filters were either known to or used within the Centre for Reviews and Dissemination (CRD), University of York, except for methods 8 and 9, which were adapted from method 7 for testing purposes. All search strategies are available in Appendix A.

**Table 1:** Methods of removing records on animals or nonhumans in Ovid Embase

Method	Application	Fields Used	Removes Animals only or Animals/Nonhumans	Origin / Usage	Citation
1	2 (animal/ or animal experiment/ or animal model/ or animal tissue/ or nonhuman/) not exp human/ 3 1 not 2	sh	Animals/Nonhumans	used within YHEC, c. 2014	[6]
2	2 (animal/ or nonhuman/) not exp human/ 3 1 not 2	sh	Animals/Nonhumans	used within CRD’s Database of Abstracts of Reviews of Effects (DARE)	[7]
3	2 animal/ or exp animal experiment/ or nonhuman/ 3 (rat or rats or mouse or mice or hamster or hamsters or animal or animals or dog or dogs or cat or cats or bovine or sheep).ti,ab,sh. 4 2 or 3 5 exp human/ or human experiment/ 6 4 not (4 and 5) 7 1 not 6	sh ti ab	Animals/Nonhumans	used within CRD’s NHS Economic Evaluations Database (NHS EED)	[7]
4	2 animal/ or exp animal experiment/ or nonhuman/ 3 (rat or rats or mouse or mice or hamster or hamsters or animal or animals or dog or dogs or cat or cats or bovine or sheep).ti,sh. 4 2 or 3 5 exp human/ or human experiment/ 6 4 not (4 and 5) 7 1 not 6	sh ti	Animals/Nonhumans	adapted from method 3	[7]
5	2 ((animal or nonhuman) not human).sh. 3 1 not 2	sh	Animals/Nonhumans	used within CCMD, c. 2021	[5]
6	2 (exp animals/ or exp animal experimentation/ or exp animal experiment/ or exp models animal/ or nonhuman/ or exp vertebrate/ or exp vertebrates/) not (exp humans/ or exp human experimentation/ or exp human experiment/) 3 1 not 2	sh	Animals/Nonhumans	Canada’s Drug Agency: Animal Studies - Standard - MEDLINE, Embase	[9]
7	2 ((exp animal/ or exp invertebrate/ or nonhuman/ or animal experiment/ or animal tissue/ or animal model/ or exp plant/ or exp fungus/) not (exp human/ or human tissue/)) 3 1 not 2	sh	Animals/Nonhumans	Embase (Excerpta Medica Database) Guide: Humans only (removes records about animals)	[10]
8	2 (exp animal/ or exp invertebrate/ or nonhuman/ or animal experiment/ or animal tissue/ or animal model/ or exp plant/ or exp fungus/) not (exp human/ or human tissue/ or human experiment/) 3 1 not 2	sh	Animals/Nonhumans	adapted from method 7 to add terms for human experiment	[10]
9	2 (exp animal/ or exp invertebrate/ or nonhuman/ or animal experiment/ or animal tissue/ or animal model/) not (exp human/ or human tissue/ or human experiment/) 3 1 not 2	sh	Animals/Nonhumans	adapted from method 7 to include terms for human experiment without plant and fungus terms	[10]
10	2 (rat or rats or mouse or mice or swine or porcine or murine or sheep or lambs or pigs or piglets or rabbit or rabbits or cat or cats or dog or dogs or cattle or bovine or monkey or monkeys or trout or marmoset$1).ti. and animal experiment/ 3 animal experiment/ not (human experiment/ or human/) 4 2 or 3 5 1 not 4	sh ti	Animals only	Glanville et al., 2019	[11]
11	2 animal experiment/ not (human experiment/ or human/) 3 1 not 2	sh	Animals only	adapted from method 10	[11]

**sh** or / = subject headings; **exp** = exploded subject heading; **ti** = title field; **ab** = abstract field; **$** = truncation.

Each record was screened by both authors using the titles and abstracts. Records were categorized as either ‘should be removed’ or ‘should be retained’, using the criteria and definitions in Table 2, with disagreements resolved through discussion. The definitions and screening criteria in Table 2 are subjective and were designed around the context of the record rather than the population, as this is the broadest and most sensitive approach. We chose this approach as definitions of animal/nonhuman studies that are ineligible and should be excluded (both in the searches and during screening) could vary depending on the review being conducted.

**Table 2:** Definitions and screening criteria

Definitions		Screening Criteria
**Animal or Nonhuman Records**	The primary focus or outcome is on, about, or for animals or nonhumans, **and** there is no relevance, impact or implication for human health (i.e., studies where humans are mentioned or compared in the study would be excluded from this categorisation if there is no relevance, impact or implication for human health). Pre-clinical studies investigating human health conditions by experimenting on animals whether wholly or in part (e.g., tissues, cells, etc), including studies also using human tissues or cells (unless human tissues, cells etc are tested separately and compared with animal tissues, cells etc). Studies that feature or use animals or nonhumans whether wholly or in part (e.g., tissues, cells, etc) with no relevance, impact or implication for human health, even if humans are mentioned or whole, living humans are compared (e.g., interns versus specialists). Studies on the properties, cultivation, use, health or diseases of nonhuman organisms such as plants, fungi, viruses, bacteria etc, where there is no relevance, impact or implication for human health.	**Should be Removed:** Meets criteria for Animal or Nonhuman records.
**Not** **Animal or Nonhuman Records**	**Anything that does not meet the definition of an animal or nonhuman record above.** Several examples include (but are not limited to): The focus is not on or about humans, animals or nonhumans (e.g., computers, the environment, etc). First-in-human studies. The primary focus or outcome is *on*, *about*, or *for* human health (except for pre-clinical studies investigating human health conditions by experimenting on animals). On, about, or involving animals/nonhumans or both animals/nonhumans and humans, where the focus or outcome is either split between humans and animals/nonhumans, or primarily about humans (e.g., training dogs to detect cancer in humans; studies of zoonotic diseases contagious to humans; nutritional value of animal products for humans).	**Should be Retained:** Meets criteria for Not Animal or Nonhuman records.

Table 1 shows 11 different methods for removing records on animals or nonhumans in Ovid Embase. Line 1 (not listed) is representative of the line the method is applied to.

### 3. Testing and Comparing the Performance of 11 Filters

To test and compare the performance of the 11 filters, we needed to find each filter’s recall of records we screened and allocated to the ‘should be removed’ or ‘should be retained’ categories. This was performed for each dataset individually.

Using the ‘an’ field code, we entered the accession numbers of each record allocated to the ‘should be removed’ and ‘should be retained’ categories with the 11 filters applied consecutively. This was performed separately for each category. The filters were applied with the NOT operator as well as with the AND operator, so we could determine the exact number of records removed or retained by each filter. Due to duplicate removal and changes to the records since their original export from Embase, some of the 3,000 records could not be retrieved using the accession number, resulting in only 2,689 records being tested overall.

For each of the 11 filters, we calculated the sensitivity, specificity, and the overall accuracy. Sensitivity, which is a measure of a filter’s ability to correctly retrieve relevant records, was calculated by dividing the number of records retained by each filter by the number of records in the ‘should be retained’ category and then multiplying this number by 100. Specificity, which is a measure of a filter’s ability to correctly exclude irrelevant records, was calculated by dividing the number of records removed by each filter by the number of records in the ‘should be removed’ category and then multiplying this number by 100. The term ‘overall accuracy’ is used to refer to the number of records that were either correctly removed or correctly retained by each filter. This was calculated by combining the number of correctly retained and removed records for each method, dividing this number by the combined number of records that should be removed or retained, and then multiplying this number by 100. Overall accuracy should be understood using the proportion of correct records in the ‘should be retained’ and ‘should be removed’ categories, as this weights the overall accuracy in favour of either sensitivity or specificity accordingly. For all calculations, percentages are shown to one decimal point.

We also sought to explore how many records with human participants or data were incorrectly excluded by each filter, using the following methodology. Across all datasets, 1,102 records in total had been categorized as ‘should be retained’. These records were then screened on title and abstract by one author to find records that used human participants or data. In total, 735 records were found for this subset, which was the most valuable to report on compared with the other reasons records should be retained (e.g., those not on animals/nonhumans or human health directly, those relevant to factors affecting human health, etc).

To find each filter’s recall of the 735 records with human participants or data, we created a search string of the accession numbers of these records with the 11 filters applied consecutively. We calculated the percentage of records removed for each filter. This was calculated using the number of records removed by each filter as proportion of the total number of records with human participants or data.

The process of testing the performance of the 11 filters is summarized in Figure 1.

**Figure 1:**
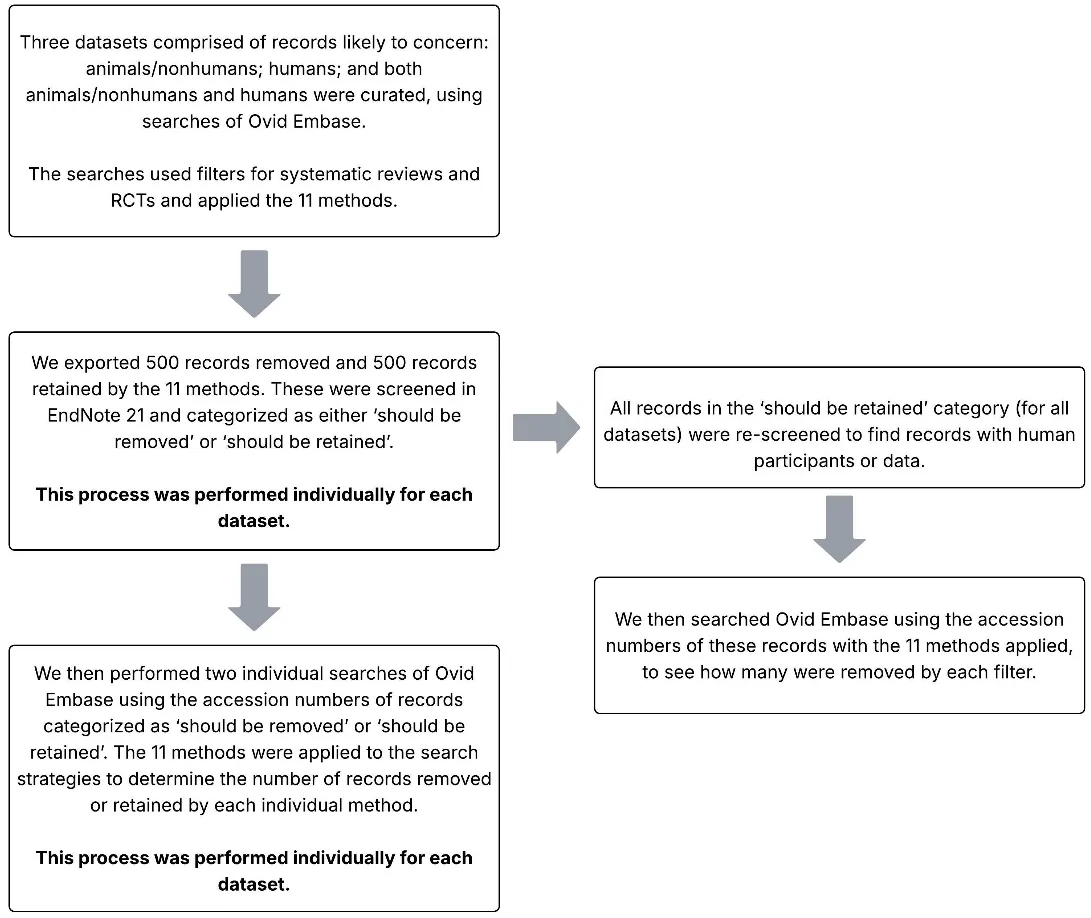
Testing the performance of 11 filters to remove records on animals or nonhumans

## RESULTS

Table 3 shows the recall of records in the ‘should be removed’ or ‘should be retained’ categories by each of the 11 filters for the humans dataset. Results with the highest percentages in each column are shown in grey and bold.

**Table 3:** Comparing the performance of 11 filters to remove records on animals or nonhumans (Humans Dataset)

Method	*Sensitivity* Should be Retained	*Specificity* Should be Removed	Overall Accuracy
1	531 of 678 (78.3%)	281 of 312 (90.1%)	812 of 990 (82%)
2	532 of 678 (78.5%)	281 of 312 (90.1%)	813 of 990 (82.1%)
3	511 of 678 (75.4%)	**286 of 312 (91.7%)**	797 of 990 (80.5%)
4	530 of 678 (78.2%)	282 of 312 (90.4%)	812 of 990 (82%)
5	531 of 678 (78.3%)	281 of 312 (90.1%)	812 of 990 (82%)
6	529 of 678 (78%)	283 of 312 (90.7%)	812 of 990 (82%)
7	520 of 678 (76.7%)	285 of 312 (91.3%)	805 of 990 (81.3%)
8	520 of 678 (76.7%)	285 of 312 (91.3%)	805 of 990 (81.3%)
9	530 of 678 (78.2%)	281 of 312 (90.1%)	811 of 990 (81.9%)
10	589 of 678 (86.9%)	251 of 312 (80.4%)	**840 of 990 (84.8%)**
11	**599 of 678 (88.3%)**	229 of 312 (73.4%)	828 of 990 (83.6%)

Method 11 was the most sensitive filter for this dataset, correctly retaining 88.3% records in the ‘should be retained’ category. A similar proportion was correctly retained by method 10 (86.9%), whereas all other filters retained similar amounts to each other, ranging from 75.4% to 78.5%.

Method 3 had highest specificity, correctly removing 91.7% records in the ‘should be removed’ category. All other filters correctly removed similar proportions of records (ranging from 90.1% to 91.3%), except for method 11 which had the lowest specificity at 73.4% and method 10 with second-lowest specificity at 80.4%.

Method 10 achieved 84.8% overall accuracy for the humans dataset. Similar percentages were observed for the other filters, which ranged from 80.5% to 83.6%.

Table 4 shows the recall of records in the ‘should be retained’ and ‘should be removed’ categories by each of the 11 filters for all datasets combined. Results with the highest percentages in each column are shown in grey and bold.

**Table 4:** Comparing the performance of 11 filters to remove records on animals or nonhumans (All Datasets)

All Datasets	Method	*Sensitivity* Should be Retained	*Specificity* Should be Removed	Overall Accuracy
**1**	868 of 1102 (78.8%)	1079 of 1587 (68%)	1947 of 2689 (72.4%)
**2**	870 of 1102 (78.9%)	1079 of 1587 (68%)	1949 of 2689 (72.5%)
**3**	829 of 1102 (75.2%)	**1134 of 1587 (71.5%)**	**1963 of 2689 (73%)**
**4**	867 of 1102 (78.7%)	1085 of 1587 (68.4%)	1952 of 2689 (72.6%)
**5**	869 of 1102 (78.9%)	1080 of 1587 (68.1%)	1949 of 2689 (72.5%)
**6**	863 of 1102 (78.3%)	1086 of 1587 (68.4%)	1949 of 2689 (72.5%)
**7**	855 of 1102 (77.6%)	1087 of 1587 (68.5%)	1942 of 2689 (72.2%)
**8**	855 of 1102 (77.6%)	1087 of 1587 (68.5%)	1942 of 2689 (72.2%)
**9**	865 of 1102 (78.5%)	1083 of 1587 (68.2%)	1948 of 2689 (72.4%)
**10**	973 of 1102 (88.3%)	792 of 1587 (49.9%)	1765 of 2689 (65.6%)
**11**	**998 of 1102 (90.6%)**	654 of 1587 (41.2%)	1652 of 2689 (61.4%)

Method 11 was the most sensitive filter for all datasets, correctly retaining 90.6% records in the ‘should be retained’ category. A similar proportion was correctly retained by method 10 (88.3%), whereas all other filters retained similar amounts to each other, ranging from 75.2% to 78.9%.

Method 3 had highest specificity, correctly removing 71.5% records in the ‘should be removed’ category. All other filters correctly removed similar proportions of records (ranging from 68% to 68.5%), except for method 11 which had the lowest specificity at 41.2% and method 10 with second-lowest specificity at 49.9%. Method 3 achieved the highest overall accuracy at 73%. Similar percentages were observed for all other filters, (ranging from 72.2% to 72.6%), except for method 11 with 61.4% overall accuracy and method 10 with 65.6%.

Table 5 shows the number of records with human participants or data removed by each of the 11 filters for all datasets combined. The percentage is based on the number of records removed as proportion of all records with human participants or data. Results with the lowest figures in each column are shown in grey and bold, with the highest figures shown in white and bold.

**Table 5:** Removed records with human participants or data (All Datasets)

Method	Records Removed
**1**	95 of 735 (12.9%)
**2**	95 of 735 (12.9%)
**3**	**105 of 735 (14.3%)**
**4**	95 of 735 (12.9%)
**5**	96 of 735 (13.1%)
**6**	96 of 735 (13.1%)
**7**	97 of 735 (13.2%)
**8**	97 of 735 (13.2%)
**9**	94 of 735 (12.8%)
**10**	102 of 735 (13.9%)
**11**	**80 of 735 (10.9%)**
**Mean**	**95.6 of 735 (13.1%)**

Method 11 removed the fewest records with human participants or data n=80 (10.9%), whereas method 3 removed the most n=105 (14.3%). The mean number of records removed was 95.6 (13.1%).

## DISCUSSION

### How do the Filters Perform and Compare?

The filters with highest sensitivity and specificity (methods 11 and 3, respectively) were the same for Table 3 (humans dataset) and Table 4 (all datasets). The samefindings were observed for each dataset individually, which can be seen in Appendix B. This suggests that the high sensitivity of method 11 and the high specificity of method 3 in comparison to the other filters, could be generalizable across a broad range of topics.

Following this pattern, the finding that the filter with the highest sensitivity (method 11) incorrectly removed the fewest records with human participants or data (n=80, 10.9%) in comparison with method 3, which removed more than any other filter (n=105, 14.3%), may be as we would expect. However, method 10, which was the second most sensitive filter overall, removed the second highest number of these records (n=102, 13.9%). This highlights the value of further insight into filter performance beyond sensitivity and specificity.

The filters which achieved the highest overall accuracy differed between the datasets. Method 10 had the highest percentage of correct records (84.8%) for Table 3 (humans dataset), whereas method 3 achieved the highest percentage (73%) for Table 4 (all datasets). This will be due to the different proportions of records in the ‘should be retained’ and ‘should be removed’ categories between the datasets, caused by the different topics that were sought (i.e., animals or nonhumans; humans; and both animals/nonhumans and humans). Although we report on the overall accuracy of the filters, records on animals/nonhumans may be unlikely to make up a significant proportion of results for reviews interested in humans as a subject or population. Therefore, we recommend choosing a filter based on sensitivity rather than overall accuracy. Where specificity is an important consideration, this is best where sensitivity also remains high. As an example, if we compare filters 10 and 11 in Table 4 (all datasets), method 10 is 2.3% less sensitive than method 11, with 8.7% higher specificity.

### Do the Different Approaches of the Filters Impact their Performance?

A key difference that affected filter performance is whether they were designed to remove records with subject headings for animals and nonhumans or for animals only. Table 1 shows that nine of the 11 methods employed subject headings to remove records on animals and nonhumans. The remaining two (methods 11 and 10) were unique in not applying the ‘nonhuman/’ subject heading. These filters, which were the two most sensitive, are also comparable in their sole use of the ‘animal experiment/’ subject heading, as ‘animal/’ appears in all other methods. Although other variations in subject heading choice can be seen across the filters, these had a much less noticeable impact on performance.

The similarities and differences in the design of the filters is reflected in the sensitivity and specificity they achieved. Although method 11 achieved the highest sensitivity at 88.3% for Table 3 (humans dataset) and 90.6% for Table 4 (all datasets), method 10 was close behind at 86.9% and 88.3%, respectively. All other filters achieved similar levels of sensitivity for both Table 3 (humans dataset) and Table 4 (all datasets).

This pattern was also observed for specificity: method 3 achieved highest specificity at 91.7% for Table 3 (humans dataset) and 71.5% for Table 4 (all datasets) but had a similar performance to most other filters, except for methods 11 and 10 which had the lowest and second-lowest specificity respectively.

Another notable difference in filter design that affected performance is the search fields. Table 1 shows that eight of the 11 methods solely employed subject headings; two employed the title field in addition to subject headings (methods 4 and 10); and one employed the title and abstract fields in addition to subject headings (method 3).

Method 3 was unique in employing the title and abstract fields in its approach to remove records. Notably, this filter had highest specificity and lowest sensitivity overall. Since terms appearing in the title and abstract fields may not necessarily reflect the content of a record, their use could have caused records to be incorrectly excluded. For example, some terms might be used to denote irrelevance (e.g., ‘animal studies were excluded’) or might be used metaphorically rather than literally, as Finnegan et al. have observed [12]. Since this filter is almost identical to method 4 but unique in its application of the abstract field, it is this aspect of its design which led to it achieving highest specificity and lowest sensitivity.

For the other two filters that employed the title field and subject headings (methods 4 and 10), performance varied. Method 4 ranked sixth for sensitivity, whereas method 10 was the second most sensitive filter overall. Additional key differences between these filters in their use of subject headings (as described above) explain this difference in performance. A further difference is that method 10 removes records with animal terms in terms in the title where these occur with the ‘animal experiment/’ subject heading. As all 11 filters remove irrelevant free-text terms or subject headings unless they occur with a relevant subject heading, method 10 is unique in its structure to remove records with irrelevant free-text terms that occur with an irrelevant subject heading. However, this also means that it is possible for method 10 to remove records with the Emtree heading ‘human/’, in contrast to all other filters. To understand the implications of this difference, this led to the removal of a first-in-human study of porcine skin xenotransplantation for burns [19] (due to having ‘porcine’ in the title and ‘animal experiment/’), and a study on autistic children’s visual processing of the faces of humans and dogs [20] (due to having ‘dog’ in the title and ‘animal experiment/’). This aspect of its design may also explain why method 10 removed the second highest number of records with human participants or data. If we compare the similar design and performance of methods 10 and 11, the unique differences in the structure of method 10 led to its correct removal of more animal records at a slight cost to sensitivity. Notably, there were only minor differences in sensitivity between methods 11 and 10 in Tables 3 and 4 (1.4% and 2.3%, respectively), with larger differences in specificity (7% and 8.7%, respectively).

Information about the origins or usage of the filters may reflect the context for which they were originally developed, which could explain some of the design differences between the filters. As an example, method 5’s use by the Cochrane Common Mental Disorders group could mean it was developed using records on common mental disorders. Similarly, method 3’s origins as part of the strategy to populate the NHS Economic Evaluations Database could mean it was developed using this study type. This might also explain aspects of its design, such as the use of the title and abstract fields, which may have been valuable for economic evaluations. In the absence of this information, it is useful to examine how the filters compare to each other for the study types used within this study (systematic reviews and RCTs).

As a further point on filter design, several Emtree headings for human study types listed in Elsevier’s 2026 indexing guide are not routinely used in filters that remove animal or nonhuman records [15]. These include: ‘normal human/’, ‘major clinical study/’, ‘clinical article/’, ‘case report/’, ‘human experiment/’, ‘human tissue/’, and ‘human cell/’ [15]. Future studies could investigate the impact of incorporating these subject headings into a new or existing filter. Although some review teams may not be interested in Emtree terms such as ‘human tissue/’ or ‘human cell/’, where *in vitro* or *ex vivo* studies are not relevant, it is worth noting that some studies may involve human tissues and cells as well as including human participants. In comparison to the filters tested in this study, a wider variety of free-text terms for humans are searched in the Embase.com search strategy used to populate Cochrane CENTRAL. Designed by Julie Glanville, the animal studies filter includes terms such as patient, patients, children, man, men, women, woman, pediatric, paediatric, and other terms [21]. This approach could be investigated for Ovid Embase or filters designed for other databases and platforms.

### How Might Indexing Affect Filter Performance?

A study by Soudant et al. reported that most of the records incorrectly excluded by animal filters in Embase were conference abstracts [14]. This finding may suggest that records which only receive automated indexing are more likely to be incorrectly excluded by animal filters. This includes the following types of record: certain non-English language records; pre-prints; conference abstracts; ‘in press’ or ‘in process’ records; records *without* one or more Emtree terms for diseases, drugs, or devices; and records *not identified as*: reviews; short surveys (for case reports only); meta-analyses; and systematic reviews. Information specialists should keep this in mind for review topics meeting these criteria (i.e., those interested in international evidence, conference abstracts, pre-prints, etc). As a result of this finding, Soudant et al. discourage the use of filters in strategies aiming to find conference abstracts [14]. However, information specialists could limit to these records (i.e., conference abstracts or pre-prints) separately in the search strategy and then use the OR operator to pool these records with results applying a filter to remove records on animals/nonhumans.

As each filter removed records with human participants or data (see Table 5), this provides evidence that records may not always be indexed as we might expect. The following examples were excluded by many (or all) of the filters as records were not indexed with ‘human/’: a study on the impact of an animal visitation program on students’ mental health [22] (missed due to indexing with ‘nonhuman/’ and ‘animal experiment/’, despite the presence of ‘university student/’); a review of drug-drug interactions of cannabis [23] (missed as cannabis is nonhuman); a trial of a vaccine in older adults [24] (missed as viruses are nonhuman); a review on smoking cessation [25] (missed as tobacco is nonhuman, despite ‘health care personnel/’ being applied); a study on monkeypox [26] (as monkeypox is nonhuman, despite ‘Bantu people/’ and ‘Aka (people)/’ being applied); and a study on canine impaction [27] incorrectly indexed as ‘nonhuman/’. These examples, which show the breadth of ‘nonhuman/’ and its relevance to human health, also highlight that records might be indexed with more specific terms for humans based on categories such as education or race instead of broader terms such as ‘human/’. This justifies the need for filters to employ a wider variety of terms for humans, such as Glanville et al.’s filter for Embase.com [21]. Further investigation is warranted to understand what factors affect the removal of relevant records (such as topics, publication types, or automated indexing) and how these could be mitigated (e.g., with different techniques for different publication types or through increasing the sensitivity of filters).

### How do we Define an Animal or Nonhuman Study?

In evidence syntheses, definitions of animal or nonhuman studies that are ineligible and should be excluded (both in the searches and during screening) could vary depending on the specific review being conducted and the level of evidence required. This suggests the need for discussions between information specialists and review teams about the types of animal or nonhuman studies they are aiming to exclude, and whether animals or nonhumans could feature in relevant studies.

In addition, review teams could consider the human records that are of interest, since various terms for humans appear in filters to retain records with these subject headings. As an example, the term ‘human experiment/’ is used for nonclinical experiments on humans such as psychological tests [15] but its nonclinical context is not immediately clear without reading the scope note. The individual meanings of subject headings is therefore an important factor in filter choice.

## LIMITATIONS

This is an exploratory study in which only 2,689 Embase records were tested. As records were retrieved using filters for systematic reviews and RCTs, filter performance could vary for other study types. As we did not intend to test differences in filter performance by study type, we did not export the same number of records for systematic reviews and RCTs. For all datasets, only titles and abstracts were double screened.

As search results on Ovid Embase are ordered by the most recent records added to the database [28], our method of exporting our sample using a selection of record numbers and ranges would have introduced some variation in the records we obtained. However, this does not reflect a truly random sample.

Screening decisions about what is or is not an ‘animal’ or ‘nonhuman’ record were subjective, and definitions could vary. For these reasons, a broad approach was taken for greater generalizability of the findings.

The additional subcategorization of records that ‘should be retained’ with ‘uses human participants or data’ was based on the titles and abstracts and performed by one author only, which could have led to minor errors.

## CONCLUSION

Between information specialists, review teams, and even on a review-by-review basis, there may be different criteria for excluding records on animals or nonhumans. Information specialists and review teams should discuss this in the context of the specific condition, phenomenon of interest, intervention, or level of evidence needed for the review. Detailed understanding of a review team’s inclusion and exclusion criteria helps ensure relevant records are not removed through filter use.

Where filter use is considered appropriate to remove records on animals or nonhumans in Ovid Embase, we recommend the use of method 11 due to its sensitivity. Our investigation of how Emtree terms are indexed suggests that performance of the filters could vary by subject, publication type, study type, and language, which information specialists should keep in mind. Some relevant records could still be removed where subject headings such as ‘human/’ or ‘human experiment/’ have not been applied. Further research could test how the filters perform for conference abstracts, pre-prints, non-English language records and subjects that are unlikely to have one or more Emtree terms for diseases, drugs, or devices. Further testing and validation of the filters for different study types, such as qualitative studies or economic evaluations, would also be useful.

Overall, our findings are informative to help information specialists understand the impact of 11 different methods in Ovid Embase to remove records on animals or nonhumans, which are compared in terms of both performance and design.

## Data Availability

Data associated with this article are available in the supplemental files or can be obtained by contacting the first author.

## SUPPLEMENTAL FILES

**Appendix A:** Search Strategies
